# Economic Assessment of High-Dose Versus Adjuvanted Influenza Vaccine: An Evaluation of Hospitalization Costs Based on a Cohort Study

**DOI:** 10.3390/vaccines9101065

**Published:** 2021-09-24

**Authors:** Robertus van Aalst, Stefan Gravenstein, Vincent Mor, Salaheddin M. Mahmud, Jan Wilschut, Maarten Postma, Ayman Chit

**Affiliations:** 1 Department of Health Sciences, University Medical Center Groningen, University of Groningen, 9700 AB Groningen, The Netherlands; m.j.postma@rug.nl; 2 Vaccine Epidemiology and Modelling, Sanofi Pasteur, Swiftwater, PA 18370, USA; Ayman.Chit@sanofi.com; 3 Department of Health Services, Policy and Practice, School of Public Health, Brown University, Providence, RI 02903, USA; stefan_gravenstein@brown.edu (S.G.); vincent_mor@brown.edu (V.M.); 4 Center of Long-Term Services and Support, Providence VA Medical Center, Providence, RI 02908, USA; 5 Center for Gerontology & Healthcare Research, Providence, RI 02903, USA; 6 Warren Alpert Medical School, Brown University, Providence, RI 02903, USA; 7 Department of Community Health Sciences, Rady College of Medicine, University of Manitoba, Winnipeg, MB R3E 0W3, Canada; Salah.Mahmud@umanitoba.ca; 8 Department of Medical Microbiology, University Medical Center Groningen, University of Groningen, 9700 AB Groningen, The Netherlands; jcwilschut@gmail.com; 9 Unit of PharmacoTherapy, -Epidemiology & -Economics (PTE2), Department of Pharmacy, University of Groningen, 9700 AB Groningen, The Netherlands; 10 Department of Economics, Econometrics & Finance, Faculty of Economics & Business, University of Groningen, 9700 AB Groningen, The Netherlands; 11 Leslie Dan Faculty of Pharmacy, University of Toronto, Toronto, ON M5S 3M2, Canada

**Keywords:** high-dose, adjuvanted, aIIV3, HD-IIV3, influenza vaccine, economic assessment

## Abstract

Two influenza vaccines are licensed in the U.S. exclusively for the 65 years and older population: a trivalent inactivated high-dose influenza vaccine (HD-IIV3) and a trivalent inactivated adjuvanted influenza vaccine (aIIV3). In a recent publication, we estimated a relative vaccine effectiveness (rVE) of HD-IIV3 vs. aIIV3 of 12% (95% CI: 3.3–20%) for influenza-related hospitalizations using a retrospective study design, but did not report the number of prevented hospitalizations nor the associated avoided cost. In this paper we report estimations for both. Methods: Leveraging the rVE of a cohort study over two influenza seasons (2016/17 and 2017/18), we collected cost data for healthcare provided to the same study population. Vaccine costs were obtained from the Medicare pricing schedule. Our economic assessment compared cost of vaccination and hospital care for patients experiencing acute respiratory or cardiovascular illness. Results: We analyzed 1.9 million HD-IIV3 and 223,793 aIIV3 recipients. Average vaccine list prices were $46.23 for HD-IIV3 and $48.26 for aIIV3. The hospitalization rates for respiratory disease in HD-IIV3 and aIIV3 recipients were 187 (95% CI: 185–189) and 212 (195–231) per 10,000 persons-years, respectively. Attributing the average cost per hospitalization of $12,652 ($12,214–$13,090) to the difference in hospitalization rates, we estimate net savings of HD-IIV3 to be $34 ($10–$62) per recipient. Conclusion: Pooled over two predominantly A/H3N2 respiratory seasons, vaccination with HD-IIV3 was associated with lower hospitalization rates and associated costs compared to aIIV3 in senior members of a large national managed health care company in the U.S. Reduced hospitalizations affect healthcare utilization overall, and therefore other costly health outcomes.

## 1. Background

Adults 65 years and older (hereinafter referred to as seniors) are at greater risk for complications following influenza infection compared with younger adults, due in part to immunosenescence and increased comorbid conditions, leading to decreased vaccine efficacy and increased severity of influenza related complications [1,2,3]. In the U.S., the annual cost of hospitalizations associated with influenza are estimated to be $1.3 billion for this age group [4]. Given this substantial cost, a health economic analysis of the various influenza vaccination strategies aiming to increase protection for this age group is pertinent.

At the time of our study, two influenza vaccines were licensed exclusively for use in seniors in the U.S.: an egg-grown trivalent inactivated high-dose influenza vaccine (Fluzone^®^ High-Dose, Sanofi Pasteur, hereinafter referred to as HD-IIV3) and an egg-grown trivalent inactivated adjuvanted influenza vaccine (Fluad^®^, Seqirus, hereinafter referred to as aIIV3). HD-IIV3 aims to improve protection through quadrupling the dose of hemagglutinin antigen (HA) per influenza strain from 15 µg HA to 60 µg HA (180 µg total), whereas aIIV3 is a standard-dose trivalent inactivated vaccine to which an oil-in-water emulsion of squalene oil adjuvant (MF59) is added. Influenza vaccines licensed to age groups including but not limited to seniors are beyond the scope of this study and at the time of writing, a quadrivalent inactivated high-dose influenza vaccine (HD-IIV4) and a quadrivalent inactivated adjuvanted influenza vaccine (aIIV4) have become available to U.S. seniors.

In a recent publication we estimated a relative vaccine effectiveness (rVE), or additional reduction, of HD-IIV3 versus aIIV3 of 12% (95% CI: 3.3–20%) for respiratory related hospitalizations; and 7% (2.3–12%) for either cardiovascular or respiratory related hospitalizations during two respiratory seasons (2016/17 through 2017/18) using a retrospective cohort study design, but did not report the number of prevented hospitalizations nor the associated avoided cost [3]. In this paper we present estimations for both. The cohort study used a statistical rate-change method (PERR) to adjust for observable and unobservable differences between the HD-IIV3 and aIIV3 recipients. The use of this method will improve the accuracy of the economic burden estimations as well [5].

## 2. Methods

### 2.1. Study Design, Population and Data Sources

Our previously published retrospective cohort study compared hospitalization rates between recipients of HD-IIV3 and aIIV3 during two respiratory seasons (2016/17 and 2017/18), using claims data from Optum’s deidentified Clinformatics^®^ Data Mart (CDM). CDM is derived from a database of administrative health claims for members of a large national managed care company affiliated with Optum (hereinafter referred to as members). Members were included when they were at least 65 years old at vaccination, had received only one HD-IIV3 or aIIV3 vaccine in the seasons of interest, and had been enrolled for at least one year before vaccination until the end of the respiratory season on 30th June. This resulted in a study population of 1.9 million HD-IIV3 and 223,793 aIIV3 recipients.

Direct comparison of the number of observed hospitalizations and associated cost between HD-IIV3 and aIIV3 recipients in this study population was not possible due to confounding factors. The cohort study, however, did report an rVE that was adjusted for measured, person-level confounding factors and unmeasured time-fixed confounding factors using the prior event rate ratio (PERR). To limit redundancy with this published study, we will not describe the PERR method and its application here in detail. In summary, vaccine exposure was ascertained by Current Procedural Terminology (CPT) codes, National Drug Codes (NDC) or brand names (Supplemental Table S1), The primary outcome was an acute hospitalization for respiratory disease, defined by its principal discharge diagnosis (International Classification of Diseases, Tenth Revision, [ICD-10]: Jxx). In addition, we reported hospitalizations for either cardiovascular or respiratory disease (ICD-10: Ixx–Jxx). Outcome rates were adjusted for the person-level confounding factors gender, race, age, Department of Health & Human Services (HHS) region (proxy for place of residency), month of vaccination, proxies for frailty, comorbid conditions, and influenza vaccination history (Supplemental Table S2). In addition, outcome rates were adjusted for time-fixed unmeasured confounding factors by comparing the outcome rate change from pre-vaccination to post-vaccination period in the HD-IIV3 cohort with the rate change in the aIIV3 cohort [6,7,8,9].

It is important, however, to realize that we used the published PERR adjusted rVEs as the starting point for our economic assessment of hospitalization costs. Modifications to the published methods and rVEs are beyond the scope of this paper.

We used a previously described method to assign the total observed hospitalizations (in both study arms of the cohort study) to the HD-IIV3 and aIIV3 recipients using the published PERR-adjusted rVEs [10] (Supplemental Analysis S1). We then calculated the absolute risk reduction (ARR) by subtracting the incidence rate in the HD-IIV3 cohort from the rate in the aIIV3 cohort. The multiplicative inverse of ARR results in the number needed to vaccinate (NNV = 1/ARR): the number of patients that need to be vaccinated with HD-IIV3 instead of aIIV3 to prevent one additional hospitalization (Table 1). To evaluate cost savings of HD-IIV3 vaccination, we estimated the difference in costs per aIIV3 recipient as if they had received HD-IIV3 instead. This was calculated as the average cost of all observed hospitalizations for aIIV3 recipients divided by the NNV minus the average cost difference of administering the two vaccines. Average hospitalization costs were calculated using the standardized costs of each of the observed hospitalizations in either cohort (Supplemental Table S4). We calculated the total realized net cost savings by multiplying the total number of HD-IIV3 recipients by the cost savings per HD-IIV3 recipient.

We obtained vaccine costs from the Medicare pricing schedule and assumed the cost of administering the vaccine to be equal for both vaccines [11]. For hospitalization cost, we obtained the standard costs from CDM for all observed hospitalizations at the patient level. The standard cost aims to remove variability in medical costs due to various reasons, including geographical location and payer negotiated contracts (Supplemental Table S4). CDM is statistically de-identified under the Expert Determination method consistent with HIPAA [12].

### 2.2. Sensitivity Analysis

The PERR method is based on the common rate-change assumption on the multiplicative scale [9]. This assumption formalizes the intuitive requirement that the rate-change in the aIIV3 group can be used as a proxy for the rate-change in the HD-IIV3 group, had they received aIIV3 instead. Because potential violations of this assumption cannot be observed in the data (untestable), we applied a sensitivity analysis that adjusts PERR with a bias parameter ranging from 0.9 to 1.1 (Supplemental Analysis S2) [9]. In addition, we added a sensitivity analysis replacing the list price of the vaccines with a scenario where HD-IIV3 is $10 more or less expensive than aIIV3, reflecting potential differences in market price.

## 3. Results

We analyzed 1,900,920 HD-IIV3 and 223,793 aIIV3 recipients. Average vaccine list prices were $46.23 for HD-IIV3 and $48.26 for aIIV3. The hospitalization rates for respiratory disease in HD-IIV3 and aIIV3 recipients were 187 (95% CI: 185–189) and 212 (195–231) per 10,000 persons-years, respectively (Table 1, Supplemental Table S3). Attributing the average cost per hospitalization of $12,652 ($12,214–$13,090) to the difference in hospitalization rates, we estimate net cost savings of $34 ($10–$62) per HD-IIV3 recipient (Table 2).

We estimate net cost savings due to reduced hospitalizations for either respiratory or cardiovascular disease of $69 ($23–$232) per HD-IIV3 recipient. Based on 1.9 million HD-IIV3 recipients, we estimate total net savings over two seasons of $65 million ($19–$119 million) due to reduced hospitalizations for respiratory disease; and $131 million ($43–$232 million) due to reduced hospitalizations for either respiratory or cardiovascular disease.

### Sensitivity Analysis

When limiting the analysis to hospitalizations for respiratory disease only, we observe greater sensitivity to magnitude of vaccine price difference compared to hospitalizations for cardiovascular or respiratory disease. When HD-IIV3 is $10 more expensive than aIIV3, the net cost savings are $22 (−$2 to $50) per HD-IIV3 recipient due to avoided respiratory hospitalizations and $108 ($20–$209) due to avoided cardiovascular or respiratory hospitalizations (Table 3). When varying the PERR bias parameter, we observe lower sensitivity in hospitalizations for respiratory disease only than in hospitalizations for either cardiovascular or respiratory disease (Table 4). Where the point estimate for net cost savings due to reduced hospitalizations for respiratory disease remains positive ($7 per HD-IIV3 recipient), the point estimate for hospitalizations for respiratory or cardiovascular disease becomes negative (−$27 per HD-IIV3 recipient).

## 4. Discussion

We analyzed 1,900,920 HD-IIV3 and 223,793 aIIV3 recipients aged 65 years and older in senior members of a large U.S. national managed care company. Pooled over two predominantly A/H3N2 respiratory seasons, HD-IIV3 was associated with lower hospitalization costs compared to aIIV3. More specifically, we estimate net cost savings of $34 ($10–$62) per HD-IIV3 recipient due to avoided hospitalizations for respiratory disease, and net cost savings due to avoided hospitalizations for either respiratory or cardiovascular disease of $69 ($23–$232) per HD-IIV3 recipient. These savings, however, are based on vaccine list prices, not necessarily reflecting market prices. In a sensitivity analysis where HD-IIV3 is $10 per dose more expensive than aIIV3, the point estimate of savings due to avoided hospitalizations for respiratory disease only stays positive, but is no longer statistically significant. For this outcome HD-IIV3 is still highly cost effective because we estimate a reduction of hospitalizations without additional cost. HD-IIV3 remains cost-saving due to avoided hospitalizations for respiratory or cardiovascular disease when $10 per dose more expensive than aIIV3.

We limited our economic assessment to the effect both vaccines had on hospitalization cost. Reduction of vaccine-preventable disease, however, is generally not limited to reduction of the most severe complications requiring hospitalization. Indeed, reduction of influenza-attributable hospitalization has been associated with reductions of influenza-attributable illness and medical visits [13,14] As a result, the total cost savings may be higher than we estimate here.

Our study is an extension of a previously published case study capturing the first two seasons that aIIV3 was available on the U.S. market, taking the reported rVEs as the starting point of our economic assessment [3]. In these first seasons, vaccination rates for the adjuvanted vaccine were much lower than for HD-IIV3 (Supplemental Table S3). It is not unreasonable to expect selection bias introduced by early adopters of aIIV3. We observed, for instance, a much higher proportion of seniors who received their aIIV3 vaccine in a community pharmacy (versus doctor’s office) compared to HD-IIV3 recipients, meaning that healthier people who have less frequent doctor’s visits were more likely to receive the adjuvanted vaccine (Supplemental Table S2). In the case study, the PERR method was applied because the authors found conventional methods (regression or conditioning on place of vaccination and all other person-level baseline characteristics) resulted in differences between the cohorts of adjusted hospitalization rates in the pre-vaccination or summer period (where similar rates were expected—a sign of residual bias caused by unmeasured confounding factors). The case study, like any retrospective study using insurance claims data, was limited in the quality of person-level data it could extract. Good proxies for confounding factors like frailty, access to care, and healthcare seeking behavior were not available. If we assume these unmeasured factors are not likely to change during the relatively short time span from pre- to post-vaccination period (i.e., they are time-fixed), the PERR method can adjust for them.

Although PERR can adjust for unmeasured time-fixed confounding factors (in addition to the conventional adjustment on all measured person-level baseline characteristics), it has important assumptions. One of the most critical is the common rate-change assumption on the multiplicative scale [9]. This assumption formalizes the intuitive requirement that the rate-change in the aIIV3 group can be used as a proxy for the rate-change in the HD-IIV3 group, had they received aIIV3 instead. In a sensitivity analysis, we tested the effect violations of this assumption has on the net cost-savings. Indeed, we observed high sensitivity to this assumption. Unfortunately, the true value of the bias parameter *u* is not observable in the data. However, it is helpful to remember from the case study that pre-treatment hospitalization rates during the summer period were 20% higher in the HD group compared with the aIIV3 group, suggesting that HD recipients were sicker or frailer than aIIV3 recipients. Going into the post-treatment period, the winter, it is not unlikely that the health status of HD recipients decreases more than the health status of aIIV3 recipients (resulting in *u* < 1). Continuing this line of thought, the estimated rVE of 12% might be an underestimation of the true rVE (Supplemental Analysis S2), and net cost savings presented in Table 4 under *u* = 1.1 would be a better estimate of the true savings [15].

Izurieta and colleagues reported that HD-IIV3 was associated with lower hospitalization rates for probable influenza (hospitalization with an administrative ICD-10 code of 489 on any position on the claim) when compared to aIIV3, with an rVE of 7.7%, (95% CI: 5.1%, 10.2%) in the 2017/18 season [16]. Using a more influenza-specific outcome—typically resulting in a higher rVE—Izurieta’s finding suggests that our PERR-adjusted rVE might be an overestimation. It is important to keep in mind, however, that Izurieta and colleagues used a propensity score method to adjust for confounding. This method cannot adjust for selection bias introduced by time-fixed unmeasured confounders. Although the authors included proxies for frailty in their model, residual confounding caused by these other unmeasured or incorrectly classified confounding factors cannot be ruled out. Given our observation that HD-IIV3 tends to be given to frailer patients than aIIV3, residual confounding would bias HD-IIV3 rVE to the null. Continuing this line of thought, the estimated rVE of 7.7% might be an underestimation of the true rVE.

Limitations of the PERR method discussed above carry over to the limitations of our economic assessment. The heterogeneity in influenza viral circulation and intensity from year to year limits the generalizability of the study results as the two seasons of our study where predominantly H3N2 with limited H1N1 or B circulation. Additionally, because our study aIIV3 cohort in 2016/17 was over fivefold smaller than in 2017/18, our combined results were heavily influenced by the second season (Supplemental Table S3). Last, small measurement errors in the ascertainment of baseline characteristics (misclassification) might explain why the stratified place of vaccination does not exactly add up to 100% (Supplemental Table S2)

A strength of our PERR analysis is that we were able to adjust for time-fixed unmeasured confounding factors, person-level measured confounding factors, and included a negative control outcome of UTI admissions where we expected no association between vaccination status and the outcome [3]. Reassuringly, we did not observe a treatment effect against UTI in the pooled analysis (Supplemental Table S3). This allows us to assume that the PERR model was not misspecified. Our sensitivity analysis of the PERR method provides reasonable arguments why the reported cost-savings are likely to be conservative. We extracted the standard cost for each observed hospitalization which better reflects the true cost of the procedures (Supplemental Table S4). To the best of our knowledge, there are no economic differences in a person hospitalized for a respiratory disease based on the vaccine received.

Our findings are substantially higher than the net savings of $1.30 per HD-IIV3 recipient due to influenza-related hospitalizations reported by Pelton and colleagues [17], the only other direct comparison of hospitalization cost we could find between HD-IIV3 and aIIV3 during a similar time period and geographic area. Important differences between the two studies may explain, in part, the different study results. First and most importantly, the outcome definitions differ in sensitivity and specificity. Second, the studies were done in different populations. Where our study was limited to a commercially insured population, the Pelton study included publicly insured subjects through Medicare. Lastly, statistical approaches between both studies differed. Notably, the Pelton study controlled for measured confounding factors using inverse probability of treatment weighting and generalized estimating equations, whereas we relied on the prior event rate ratio method that controls for both measured and unmeasured confounding factors.

In the 2020/21 season, a quadrivalent inactivated high-dose influenza vaccine (HD-IIV4) and a quadrivalent inactivated adjuvanted influenza vaccine (aIIV4) will be available for U.S. seniors. As the use of the HD-IIV4 and aIIV4 grows, future studies should attempt to examine costs associated with more specific outcomes such as hospital admissions with a principal discharge code for pneumonia/influenza and hospital admissions following a positive influenza test. These specific endpoints will take public health closer to understanding the causal link between these vaccines and costs avoided due to preventing adverse health outcomes following an influenza infection.

In addition, we believe that policy makers would benefit from scientific standards for comparative vaccine effectiveness studies that aim to improve the comparability of those studies, and the detection and magnitude of residual bias in the reported results.

## 5. Conclusions

Pooled over two predominantly A/H3N2 respiratory seasons, HD-IIV3 was associated with lower hospitalization costs compared to aIIV3 in senior members of a large national managed health care company in the U.S. Reduced hospitalizations affect healthcare utilization overall, and therefore other costly health outcomes.

## Figures and Tables

**Table 1:** Number of influenza vaccinations, hospitalization rates for respiratory or cardio-vascular disease, and number needed to vaccinate (NVV) to prevent one hospitalization for members of members of large national managed health care company in the U.S. during respiratory seasons 2016/17 through 2017/18. All rates are per 10,000 person-years.

Study Cohort *			2,124,713	
HD-IIV3 recipients			1,900,920	89%
aIIV3 recipients			223,793	11%
Observed hospitalizations in study cohort *				
Respiratory			40,235	
Respiratory or cardio-vascular			119,509	
Applied relative vaccine effectiveness (rVE) *				
Respiratory			12% (3.3–20%)	
Respiratory or cardio-vascular			7% (2.3–12%)	
Hospitalizations for respiratory disease				
Rate HD-IIV3			187 (185–189)	
Rate aIIV3			212 (195–231)	
Hospitalizations for respiratory or cardio-vascular disease				
Rate HD-IIV3			558 (555–561)	
Rate aIIV3			600 (574–628)	
Absolute Risk Reduction (ARR)				
Respiratory			25 (6–46)	
Respiratory or cardio-vascular			42 (18–68)	
Number Needed to Vaccinate (NNV)				
Respiratory			393 (217–1553)	
Respiratory or cardio-vascular			238 (136–758)	

* Previously published [3].

**Table 2:** Relative vaccine effectiveness, number needed to vaccinate (NNV), and net cost savings for members of a large national managed health care company in the U.S. during respiratory seasons 2016/17 through 2017/18.

				Net Cost Savings	
Hospitalization	rVE *	NNV	Hospitalization Cost in USD	Per HD Recipient	Two Seasons Total in Million USD
Respiratory	12% (3.3–20%)	393 (217–1553)	12,652 (12,214–13,090)	34 (10–62)	65 (19–119)
Cardiovascular or Respiratory	7% (2.3–12%)	238 (136–758)	15,956 (15,618–16,294)	69 (23–232)	131 (43–232)

* Previously published [3]. Net cost savings per HD recipient are calculated by dividing the average cost for one hospitalization in the aIIV3 cohort by the number needed to vaccinate (NNV) minus the average cost difference of administering the two vaccines ($ −2.03).

**Table 3:** Sensitivity analysis of the effect of a 10 USD vaccination price difference on the net cost savings.

	Net Cost Savings: Base Case		Net Cost Savings: HD-IIV3 $10 More Expensive		Net Cost Savings: aIIV3 $10 More Expensive	
Hospitalization	Per HD Recipient in USD	Two Seasons Total in Million USD	Per HD Recipient in USD	Two Seasons Total in Million USD	Per HD Recipient in USD	Two Seasons Total in Million USD
Respiratory	34 (10–62)	65 (19–119)	22 (−2 to 50)	42 (−4 to 96)	42 (18–70)	80 (34–134)
Cardiovascular or Respiratory	69 (23–232)	131 (43–232)	57 (11–110)	108 (20–209)	77 (31–130)	146 (58–247)

**Table 4:** Sensitivity analysis net cost savings: PERR bias parameter +/− 10% of base case (*u* = 1).

	Net Cost Savings: Base Case (*u* = 1)		Net Cost Savings: *u* = 0.9 (−10%)		Net Cost Savings: *u* = 1.1 (+10%)	
Hospitalization	Per HD Recipient in USD	Two Seasons Total in Million USD	Per HD Recipient in USD	Two Seasons Total in Million USD	Per HD Recipient in USD	Two Seasons Total in Million USD
Respiratory	34 (10–62)	65 (19–119)	7 (−14 to 35)	14 (−27 to 66)	60 (33–94)	115 (64–179)
Cardiovascular or Respiratory	69 (23–232)	131 (43–232)	−27 (−72 to 25)	−51 (−137 to 48)	163 (115–216)	310 (219–411)

If one believes the base case overestimates the net cost savings because of violations of the common rate ratio assumption, values of (*u* < 1) will provide a better estimate (and vice versa).

## Data Availability

The data underlying the results presented in the study contains proprietary elements owned by Optum and, therefore, cannot be broadly disclosed or made publicly available. This data can, however, be made available to a third party via the corresponding author upon reasonable request assuming certain data security and privacy protocols are in place and that the third party has executed Optum’s standard license agreement which includes restrictive covenants governing the use of the data.

## Supplementary Materials

The following are available online at https://www.mdpi.com/article/10.3390/vaccines9101065/s1, Table S1: Ascertainment of treatment, Table S2: Baseline characteristics used in the PERR-adjustment of the relative vaccine effectiveness (rVE) of HD-IIV3 versus aIIV3, Table S3: Relative vaccine effectiveness (rVE), incidence rates, absolute risk reduction (ARR) and number needed to vaccinate (NNV), Table S4: Mean standard cost and median length of stay of a hospitalization for respiratory disease, cardio-respiratory disease, or urinary tract infection (UTI). Cost reported with 95% confidence intervals (CI) and length of stay (LOS) with 25th and 75th percentiles. Analysis S1: Estimating the number of hospitalizations, Analysis S2: Sensitivity analysis of the PERR method.

## References

[1] Lambert N.D., Ovsyannikova I.G., Pankratz V.S., Jacobson R.M., Poland G.A. (2012). Understanding the immune response to seasonal influenza vaccination in older adults: A systems biology approach. Expert Rev. Vaccines.

[2] Garten R., Blanton L., Elal A.I.A., Alabi N., Barnes J., Biggerstaff M., Brammer L., Budd A.P., Burns E., Cummings C.N. (2018). Update: Influenza activity in the United States during the 2017–18 season and composition of the 2018–2019 influenza vaccine. Morb. Mortal. Wkly. Rep..

[3] van Aalst R., Gravenstein S., Mor V., Mahmud S.M., Wilschut J., Postma M., Chit A. (2020). Comparative effectiveness of high dose versus adjuvanted influenza vaccine: A retrospective cohort study. Vaccine.

[4] Putri W.C., Muscatello D.J., Stockwell M.S., Newall A.T. (2018). Economic burden of seasonal influenza in the United States. Vaccine.

[5] Tannen R.L., Weiner M.G., Xie D. (2008). Replicated studies of two randomized trials of angiotensin-converting enzyme inhibitors: Further empiric validation of the ‘prior event rate ratio’ to adjust for unmeasured confounding by indication. Pharmacoepidemiol. Drug Saf..

[6] Lin N.X., Henley W.E. (2016). Prior event rate ratio adjustment for hidden confounding in observational studies of treatment effectiveness: A pairwise Cox likelihood approach. Stat. Med..

[7] Tannen R.L., Weiner M.G., Xie D. (2009). Use of primary care electronic medical record database in drug efficacy research on cardiovascular outcomes: Comparison of database and randomised controlled trial findings. BMJ.

[8] Yu M., Xie D., Wang X., Weiner M.G., Tannen R.L. (2012). Prior event rate ratio adjustment: Numerical studies of a statistical method to address unrecognized confounding in observational studies. Pharmacoepidemiol. Drug Saf..

[9] Van Aalst R., Thommes E.W., Postma M., Chit A., Dahabreh I.J. (2020). On the causal interpretation of rate-change methods: The prior event rate ratio and rate difference (in press). Am. J. Epidemiol..

[10] van Aalst R., Russo E.M., Neupane N., Mahmud S.M., Mor V., Wilschut J., Chit A., Postma M., Young-Xu Y. (2019). Economic assessment of a high-dose versus a standard-dose influenza vaccine in the US Veteran population: Estimating the impact on hospitalization cost for cardio-respiratory disease. Vaccine.

[11] Centers for Medicare and Medicaid Services Fee Schedules—General Information. https://www.cms.gov/Medicare/Medicare-Fee-for-Service-Payment/FeeScheduleGenInfo/index.

[12] U.S. Department of Health and Human Services Guidance Regarding Methods for De-Identification of Protected Health Information in Accordance with the Health Insurance Portability and Accountability Act (HIPAA) Privacy Rule 2012, Paragraph §164.514(b). https://www.hhs.gov/hipaa/for-professionals/privacy/special-topics/de-identification/index.html.

[13] Young-Xu Y., Van Aalst R., Mahmud S.M., Rothman K.J., Snider J.T., Westreich D., Mor V., Gravenstein S., Lee J.K.H., Thommes E.W. (2018). Relative Vaccine Effectiveness of High-Dose Versus Standard-Dose Influenza Vaccines Among Veterans Health Administration Patients. J. Infect. Dis..

[14] Rolfes M.A., Flannery B., Chung J.R., O’Halloran A., Garg S., Belongia E.A., Gaglani M., Zimmerman R.K., Jackson M.L., Monto A.S. (2019). Effects of influenza vaccination in the United States during the 2017–2018 influenza season. Clin. Infect. Dis..

[15] Ainslie K.E., Haber M., Orenstein W.A. (2019). Challenges in estimating influenza vaccine effectiveness. Expert Rev. Vaccines.

[16] Izurieta H.S., Chillarige Y., Kelman J., Wei Y., Lu Y., Xu W., Lu M., Pratt D., Chu S., Wernecke M. (2019). Relative effectiveness of cell-cultured and egg-based influenza vaccines among the U.S. elderly, 2017–2018. J. Infect. Dis..

[17] Pelton S.I., Divino V., Shah D., Mould-Quevedo J., DeKoven M., Krishnarajah G., Postma M.J. (2020). Evaluating the Relative Vaccine Effectiveness of Adjuvanted Trivalent Influenza Vaccine Compared to High-Dose Trivalent and Other Egg-Based Influenza Vaccines among Older Adults in the US during the 2017–2018 Influenza Season. Vaccines.

