# Supplementary material for: Economic Assessment of High-Dose Versus Adjuvanted Influenza Vaccine: An Evaluation of Hospitalization Costs Based on a Cohort Study
Source: Vaccines (Basel). 2021 Sep 24;9(10):1065. doi: 10.3390/vaccines9101065 (PMC8540428; doi:10.3390/vaccines9101065)
Supplement: Supplementary file 1 [file vaccines-09-01065-s001.zip › vaccines-1294011-supplementary.pdf]

Tables

Supplemental Table S1. Ascertainment of treatment

Supplemental Table S2. Baseline characteristics of high dose (HD-IIV3) and adjuvanted influenza vaccine (aIIV3) recipients

Supplemental Table S3. Relative vaccine effectiveness (rVE), incidence rates, absolute risk reduction (ARR) and number needed to vaccinate (NNV)

Supplemental Table S4: Mean standard cost and median length of stay of a hospitalization for respiratory disease, cardio-respiratory disease, or urinary tract infection (UTI). Cost reported with 95% confidence intervals (CI) and length of stay (LOS) with 25th and 75th percentiles.

Analyses

Supplemental Analysis S1. Application of the PERR method

Figure S1. Previous Event Rate Ratio (PERR) for season 2017-18: Hospitalizations for respiratory disease

Supplemental Analysis S2. Discussion of the PERR method

Figure S2. PERR of HD vs. aIIV3 for different values of the bias parameter *u* with 95% confidence intervals

Supplemental Table S1. Ascertainment of treatment

Vaccinations received in a doctor's office were identified by Current Procedural Terminology (CPT) codes or National Drug Code (NDC). Vaccinations received in a community pharmacy were identified by brand names.

	CPT	NDC	VAC DATE
FLUZONE HIGH-DOSE 2016-2017	90662	49281039965<br>49281039988	>= '2016-07-01'<br>< '2017-07-01
FLUAD 2016-2017	90653	70461000101<br>70461000111	
FLUZONE HIGH-DOSE 2017-2018	90662	49281040188<br>49281040165	>= '2017-07-01'<br>< '2018-07-01
FLUAD 2017-2018	90653	70461000211<br>70461000201	

NDCs are not always updated in a timely fashion, especially in the inpatient or outpatient setting, and were verified by vaccination date.

Supplemental Table S2. Baseline characteristics of high dose (HD-IIV3) and adjuvanted influenza vaccine (aIIV3) recipients

	Season 2016/17					Season 2017/18				
	HD-IIV3		aIIV3		SMD*	HD-IIV3		aIIV3		SMD*
Study Population	842,282		34,157			1,058,638		189,636		
**Gender**										
Male	351,264	42%	14,474	42%	-0.01	443,049	42%	79,031	42%	0.00
Female	490,969	58%	19,683	58%	0.01	615,545	58%	110,596	58%	0.00
Unknown	49					44		9		
**Race**										
Asian	23,751	2.8%	1,000	2.9%	-0.01	29,845	2.8%	4,801	2.5%	0.02
African American	54,599	6.5%	1,896	5.6%	0.04	75,833	7.2%	15,011	7.9%	-0.03
Hispanic	61,328	7.3%	2,296	6.7%	0.02	76,975	7.3%	11,645	6.1%	0.05
White	569,019	68%	23,351	68%	-0.02	689,639	65%	124,306	66%	-0.01
Unknown Race	138,384	16%	5,728	17%	-0.01	168,024	16%	30,818	16%	-0.01
**Age**										
65-69	189,369	22%	8,096	24%	-0.03	233,431	22%	42,784	23%	-0.01
70-74	234,809	28%	10,060	29%	-0.03	307,216	29%	57,242	30%	-0.03
75-79	175,214	21%	7,116	21%	0.00	227,916	22%	40,576	21%	0.00
80-84	125,263	15%	4,736	14%	0.03	150,231	14%	25,772	14%	0.02
85+	117,627	14%	4,149	12%	0.05	139,844	13%	23,262	12%	0.03
Age (mean, sd)	75.57	6.67	75.14	6.49	0.07	75.46	6.71	75.22	6.61	0.04
**HHS Region**										
Region 1: CT, ME, MA, NH, RI, VT	31,004	3.7%	3,437	10%	-0.25	40,951	3.9%	8,004	4.2%	-0.02
Region 2: NJ, NY, PR, VI	62,302	7.4%	2,993	8.8%	-0.05	72,956	6.9%	15,392	8.1%	-0.05
Region 3: DE, DC, MD, PA, VA, WV	27,988	3.3%	619	1.8%	0.10	33,411	3.2%	4,737	2.5%	0.04
Region 4: AL, FL, GA, KY, MS, NC, SC, TN	210,553	25%	7,359	22%	0.08	251,396	24%	79,399	42%	-0.39
Region 5: IL, IN, MI, MN, OH, WI	165,564	20%	6,221	18%	0.04	204,987	19%	26,293	14%	0.15
Region 6: AR, LA, NM, OK, TX	83,243	10%	3,193	9.3%	0.02	118,510	11%	10,684	5.6%	0.20
Region 7: IA, KS, MO, NE	38,421	4.6%	1,177	3.4%	0.06	57,121	5.4%	4,040	2.1%	0.17
Region 8: CO, MT, ND, SD, UT, WY	59,664	7.1%	3,159	9.2%	-0.08	74,882	7.1%	7,596	4.0%	0.13
Region 9: AZ, CA, GU, HI, NV	122,521	15%	3,773	11%	0.10	155,834	15%	22,016	12%	0.09
Region 10, AK, ID, OR, WA	39,264	4.7%	2,151	6.3%	-0.07	46,356	4.4%	11,108	5.9%	-0.07
Unknown Region	1,758	0.2%	75	0.2%	0.00	2,234	0.2%	367	0.2%	0.00

	Season 2016/17					Season 2017/18				
	HD-IIV3		aIIV3		SMD*	HD-IIV3		aIIV3		SMD*
Study Population	842,282		34,157			1,058,638		189,636		
**Month of Vaccination**										
August & September	298,370	35%	7,009	21%	0.34	354,103	33%	60,234	32%	0.04
October	335,082	40%	9,497	28%	0.26	437,160	41%	77,418	41%	0.01
November	130,471	15%	8,711	26%	-0.25	160,983	15%	28,144	15%	0.01
December & January	68,463	8.1%	7,886	23%	-0.42	91,407	8.6%	20,429	11%	-0.07
Other	9,896	1.2%	1,054	3.1%	-0.13	14,985	1.4%	3,411	1.8%	-0.03
**Time at risk**										
Baseline period (mean, sd)	107	33	129	41	-0.58	108	35	110	39	-0.05
Observation period (mean, sd)	242	35	221	42	0.56	241	37	239	40	0.04
**Point of Vaccination**										
Community Pharmacy	413,339	49%	24,277	71%	-0.46	513,661	49%	135,577	71%	-0.48
Doctor's office	440,955	52%	10,444	31%	0.45	564,019	53%	56,924	30%	0.49
**Frailty Proxy**										
No hospitalization record found	743,299	88%	30,458	89%	-0.03	930,074	88%	168,412	89%	-0.03
All-cause hospitalizations (mean, sd)	0.19	0.64	0.17	0.60	0.03	0.19	0.66	0.17	0.61	0.03

	Season 2016/17					Season 2017/18				
	HD-IIV3		aIIV3		SMD*	HD-IIV3		aIIV3		SMD*
Study Population	842,282		34,157			1,058,638		189,636		
**Comorbid Conditions**										
No record of comorbid conditions found	401,006	48%	17,782	52%	-0.09	482,522	46%	90,969	48%	-0.05
Myocardial Infarction	23,500	2.8%	864	2.5%	0.02	32,502	3.1%	5,284	2.8%	0.02
Congestive Heart Failure	60,669	7.2%	2,094	6.1%	0.04	84,497	8.0%	13,278	7.0%	0.04
Peripheral Vascular Disease	77,503	9.2%	2,721	8.0%	0.04	107,632	10%	17,441	9.2%	0.03
Cerebrovascular Disease	52,437	6.2%	1,981	5.8%	0.02	69,223	6.5%	11,869	6.3%	0.01
Dementia	25,859	3.1%	1,040	3.0%	0.00	34,905	3.3%	5,638	3.0%	0.02
Chronic Pulmonary Disease	107,899	13%	3,970	12%	0.04	146,273	14%	24,726	13%	0.02
Connective Tissue / Rheumatic Disease	22,506	2.7%	809	2.4%	0.02	29,583	2.8%	5,055	2.7%	0.01
Peptic Ulcer Disease	5,230	0.6%	178	0.5%	0.01	6,778	0.6%	1,207	0.6%	0.00
Mild Liver Disease	14,929	1.8%	581	1.7%	0.01	21,396	2.0%	3,732	2.0%	0.00
Diabetes without complications	193,794	23%	7,046	21%	0.06	258,211	24%	43,469	23%	0.03
Diabetes with complications	81,327	10%	2,786	8.2%	0.05	121,912	12%	19,301	10%	0.04
Paraplegia and Hemiplegia	3,958	0.5%	145	0.4%	0.01	5,738	0.5%	873	0.5%	0.01
Renal Disease	102,056	12%	3,437	10%	0.07	138,137	13%	22,069	12%	0.04
Cancer	76,567	9.1%	2,926	8.6%	0.02	96,641	9.1%	16,965	8.9%	0.01
Moderate or Severe Liver Disease	1,407	0.2%	54	0.2%	0.00	2,015	0.2%	327	0.2%	0.00
Metastatic Carcinoma	6,897	0.8%	248	0.7%	0.01	9,323	0.9%	1,468	0.8%	0.01
AIDS/HIV	579	0.1%	18	0.1%	0.01	891	0.1%	162	0.1%	0.00
Deyo-Charlson Score (mean, sd)	1.38	1.94	1.22	1.82	0.08	1.50	2.06	1.38	1.96	0.06
**Vaccinated in previous season**										
No vaccination record found	155,647	18%	7,568	22%	-0.09	221,972	21%	40,370	21%	-0.01
HD-IIV3	501,035	59%	17,464	51%	0.17	655,102	62%	105,145	55%	0.13
aIIV3	19	0.0%	4	0.0%	-0.01	17,423	1.6%	12,007	6.3%	-0.24
SD-IIV3	110,739	13%	6,524	19%	-0.16	79,540	7.5%	18,816	9.9%	-0.09
SD-IIV4	73,769	8.8%	2,494	7.3%	0.05	82,138	7.8%	12,217	6.4%	0.05
Other vaccine	1,073	0.1%	103	0.3%	-0.04	2,463	0.2%	1,081	0.6%	-0.05

HD-IIV3: high dose, trivalent; aIIV3: adjuvanted, trivalent; SD-IIV3: standard dose, trivalent; SD-IIV4: standard dose, quadrivalent; Other vaccine: cell culture-based, quadrivalent; recombinant, quadrivalent; live-attenuated, quadrivalent.

*Common characteristics between the HD-IIV3 and aIIV3 cohorts with an absolute standardized mean difference (SMD) of more than or equal to 0.10 suggests a substantial difference in proportions between groups.

Supplemental Table S3. Relative vaccine effectiveness (rVE), incidence rates, absolute risk reduction (ARR) and number needed to vaccinate (NNV)

		Rate per 10,000 person-years			
**Hospitalization**	**rVE**	**HD-IIV3**	**aIIV3**	**ARR**	**NNV**
Respiratory	12% (3.3% – 20%)	187 (185 – 189)	212 (195 – 231)	25 (6 – 46)	393 (217 – 1,553)
Cardiovascular or Respiratory	7% (2.3% – 12%)	558 (555 – 561)	600 (574 – 630)	42 (18 – 68)	238 (147 – 561)
Urinary tract infection (UTI)	-0.7% (-14% to 13%)	60 (59 – 61)	60 (54 – 68)	0 (-7 to 9)	

NNV: number of patients that need to be vaccinated with HD-IIV3 instead of aIIV3 to prevent one additional hospitalization.

UTI: Urinary tract infection is a negative control outcome: a treatment effect is not expected nor observed

Supplemental Table S4: Mean standard cost and median length of stay of a hospitalization for respiratory disease, cardio-respiratory disease, or urinary tract infection (UTI). Cost reported with 95% confidence intervals (CI) and length of stay (LOS) with 25th and 75th percentiles.

**Hospitalization**	**Cost**		**LOS**	
	**HD** Mean (95% CI)	**Fluad** Mean (95% CI)	**HD** Median (25th – 75th)	**Fluad** Median (25th – 75th)
Respiratory	12,351 (12,189 – 12,513)	12,652 (12,214 – 13,090)	4 (3 – 8)	5 (3 – 8)
Cardiorespiratory	15,284 (15,170 – 15,399)	15,956 (15,618 – 16,294)	4 (2 – 8)	4 (2 – 8)
UTI	12,421 (12,175 – 12,668)	12,984 (12,292 – 13,676)	4 (2 – 9)	4 (3 – 9)

Standard cost

Optum does not provide researchers the true reimbursed amount of a specific healthcare claim (charge) because it 1) it doesn't fully comply with HIPAA deidentification requirements, and 2) it doesn't necessarily reflect the true cost of the procedure as they are subject to discounts or negotiated contracts between providers and plans. Instead, Optum provides standard cost for each claim.

Standard cost aims to remove variability in medical costs due to various reasons including geographical location and payer negotiated contracts. Medical costs for the same procedure in the same year can vary widely across the country.

The methodology Optum uses to derive standard cost is based on data aggregated over the previous year of adjudicated commercial insurance claims. Facility Inpatient, Facility Outpatient, Professional and Pharmacy services all have separate algorithms to specifically price each type of care. Three of the algorithms start with a framework used by CMS for pricing Medicare services, based on DRG, procedure code or revenue code. Then Optum uses its large database of actual commercial costs to adjust the standard cost based on what our data shows for the previous year. The actual commercial costs are evaluated each year and the standard costs are adjusted accordingly.

Optum developed standard costs algorithms to address the need for researchers to systematically evaluate direct healthcare costs: facilities, professional services, pharmacy. Healthcare charges aren't an accurate reflection of either the provider or payer costs. Similarly, actual reimbursements aren't a consistent measure of actual costs as they are subject to discounts or negotiated contracts between the providers and plans. Commercial contract terms produce different unit costs for providers of the same service. These vary from geographic area to geographic area and from year to year, leading to further variabilities when assessing health care costs over time. For these reasons, comparisons of reimbursement data without adjustment for these factors can lead to inaccurate findings and conclusions.

Supplemental Analysis S1. Application of the PERR method

We used the same population and methods previously published by Van Aalst et al. to calculate the number of additionally prevented hospitalizations attributable to HD-IIV3 vaccination [1]. Please find a copy below for your convenience.

We are interested in comparing the effectiveness of the HD-IIV3 and aIIV3 vaccines. Because we expected confounding by indication – resulting in treatment selection bias – by variables that are either unmeasured or measured inaccurately (e.g. baseline comorbidities), we employed the previous event rate ratio (PERR) approach, which adjusts for measured and unmeasured, time-fixed confounding factors [2-4]. This approach, a type of difference-in-differences analysis [5], compares the outcome rate change from baseline to observation period in the HD-IIV3 cohort with the rate change in the aIIV3 cohort (Supplemental Figure 1). These rate changes can be rewritten as the change in the relative risk from baseline ($\mathrm{RR_b}$) to observation period ($\mathrm{RR_o}$), or $(\frac{\mathrm{RR_o}}{\mathrm{RR_b}})$: a measure of the treatment effect adjusted for unmeasured time-fixed confounding variables (variables that are constant during the baseline and observation periods of a given respiratory season). We selected the PERR method because its performance to reduce bias caused by unmeasured confounding factors has been thoroughly described, both in simulation studies [2, 4, 6] and an empirical study comparing PERR estimates with RCTs [7]. The crude (unadjusted for measured baseline variables) relative vaccine effectiveness (rVE) is calculated as

$$rVE = \left(1 - \frac{RR_o}{RR_b}\right) \times 100\% \qquad \text{(S1)}$$

We estimated crude rVEs by fitting a Poisson regression model with an interaction term between two variables, the $period$ (observation versus baseline) and the $treatment$ (HD-IIV3 versus aIIV3). The regression model is shown below.

$$\begin{aligned} \log\big(E(Y)\big) = {} & season + period + treatment + \\ & season \times period + season \times treatment + period \times treatment + \\ & season \times period \times treatment + \log(\text{days at risk}) \qquad \text{(S2)} \end{aligned}$$

The coefficient of the interaction term $period \times treatment$ is used to estimate $\left(\frac{RR_o}{RR_b}\right)$, and thus the crude rVE. We adjusted the crude rVE for measured confounders by adding all baseline characteristics of Supplemental Table 2 as covariates to the model above (model 2, except for Age Groups and the Deyo-Charlson Score, to prevent collinearity with Age and individual comorbid conditions). Pooled results over the two seasons were also calculated by removing

the interaction term $season \times period \times treatment$. We used a robust variance estimator for each rVE, and after exponentiation, the delta-method to calculate 95% confidence intervals.

We compared baseline characteristics between HD-IIV3 and aIIV3 recipients using standardized mean differences. We adopted the rule that an absolute standardized mean difference smaller than 0.1 suggests no substantial difference between the compared groups [8].

Figure S1. Previous Event Rate Ratio (PERR) for season 2017-18: Hospitalizations for respiratory disease

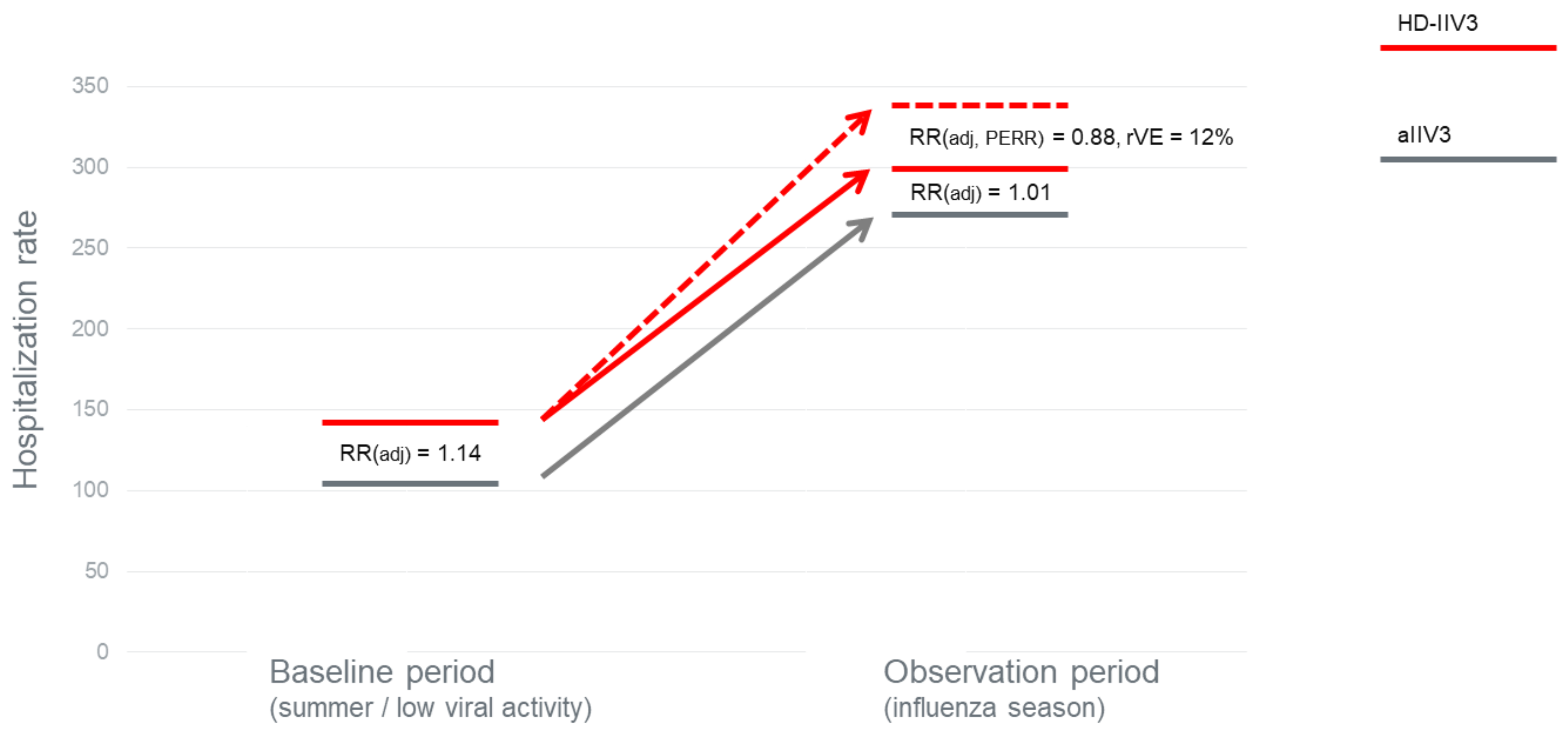

We used outcome rates in the baseline period as an indicator for confounding. This is the summer period when there was no, or very low viral activity in the U.S. We observed almost 30% higher respiratory-related hospitalization rates in the HD-IIV3 recipients in the baseline period, suggesting that aIIV3 recipients were a bit healthier than HD-IIV3 recipients. After adjusting for all the baseline characteristics, the relative risk reduced to 1.14, so closer to 1. However, if we were able to adjust for all confounding, we would expect the relative risk to be 1 in this period. It is important to realize this, because if we only adjusted the hospitalization rates in the observation period for baseline characteristics, the estimated rVE would have a similar residual confounding.

PERR adjusts for this type of residual confounding in the following way:

- The hospitalization rates in the aIIV3 recipients increased almost a factor two and a half from baseline to observation period (grey arrow)
- IF the HD-IIV3 recipients had received the aIIV3 vaccine instead, we would expect their rates to increase with the same magnitude, a factor two and a half as well (red dotted arrow)
- However, we observed lower rates (red solid arrow). After adjusting the rates in the observation period with all the baseline characteristics and applying PERR we saw 12% lower rates than expected
- Had we not adjusted for unmeasured confounding factors in this way, we would have seen no treatment effect. RR(adj) = 1.01

Supplemental Analysis S2. Discussion of the PERR method

The common rate-change assumption on the multiplicative scale (common rate ratio assumption) – formalizes the intuitive requirement that the ratio of the counterfactual post- and pre-treatment rates among the treated under intervention to administer the control strategy equals the ratio of the factual post- and pre-treatment rates among the control group [9].

The common rate-change assumption is violated when time-varying factors have an unequal effect on the treatment and control group. If important patient characteristics or comorbid conditions turn out to be less "time-fixed" than assumed (e.g. smoking status, frailty), and change more - from pre-treatment to post-treatment - in the control group than the treatment group (or vice versa), the rate-change in the control group can no longer be used as a proxy for the counterfactual rate-change in the treated, had treatment been withheld. This assumption is also violated when heterogeneity of exposure to the influenza virus cannot be ruled out. Because viral activity is regionally dependent, a potential (and often applied) solution is to match the cohorts on location of vaccination. One of the limitations of using insurance claims data is the limited geographic granularity.

Because this assumption can be violated in many ways, we examined how our conclusions might be affected under different "degrees of assumption violation", or values of bias parameter *u*. To illustrate this method, Figure 2 shows the sensitivity analysis for the HD vs. aIIV3 study. Because the bias parameter *u* is not identifiable, our estimate is biased (under or over estimating the treatment effect), we chose a range of +/- 0.2 for *u* (*u*=1 equals the published estimate or base case, a pooled rVE of 12% (95% CI: 3.3% - 20%) to allow for a sign switch of the treatment effect.

The main limitation of this sensitivity analysis is that we don't know which value of *u* the true value is, or what a realistic range would be: further research is necessary to address this question. Intuitively, we can say that small violations (in either direction) will keep the treatment effect of HD versus aIIV3 positive, but when there are major violations, the direction does matter. A bias parameter smaller than 0.97 results in a non-significant treatment effect. In other words, if HD recipients are more likely to decrease their health status from pre to post period compared to aIIV3 recipients, PERR underestimates the treatment effect. Here, it is helpful to remember that baseline hospitalization rates during the summer period were 20% higher in the HD group compared with the aIIV3 group, suggesting that HD recipients were *sicker or frailer* than aIIV3 recipients. Going into the post-treatment period, the winter, it is not unlikely that the health status of HD recipients decreases more than the health status of aIIV3 recipients (resulting in $u < 1$). Continuing this line of thought, the estimated rVE of 12% is likely to be an underestimation of the true rVE.

Figure S2. PERR of HD vs. aIIV3 for different values of the bias parameter *u* with 95% confidence intervals

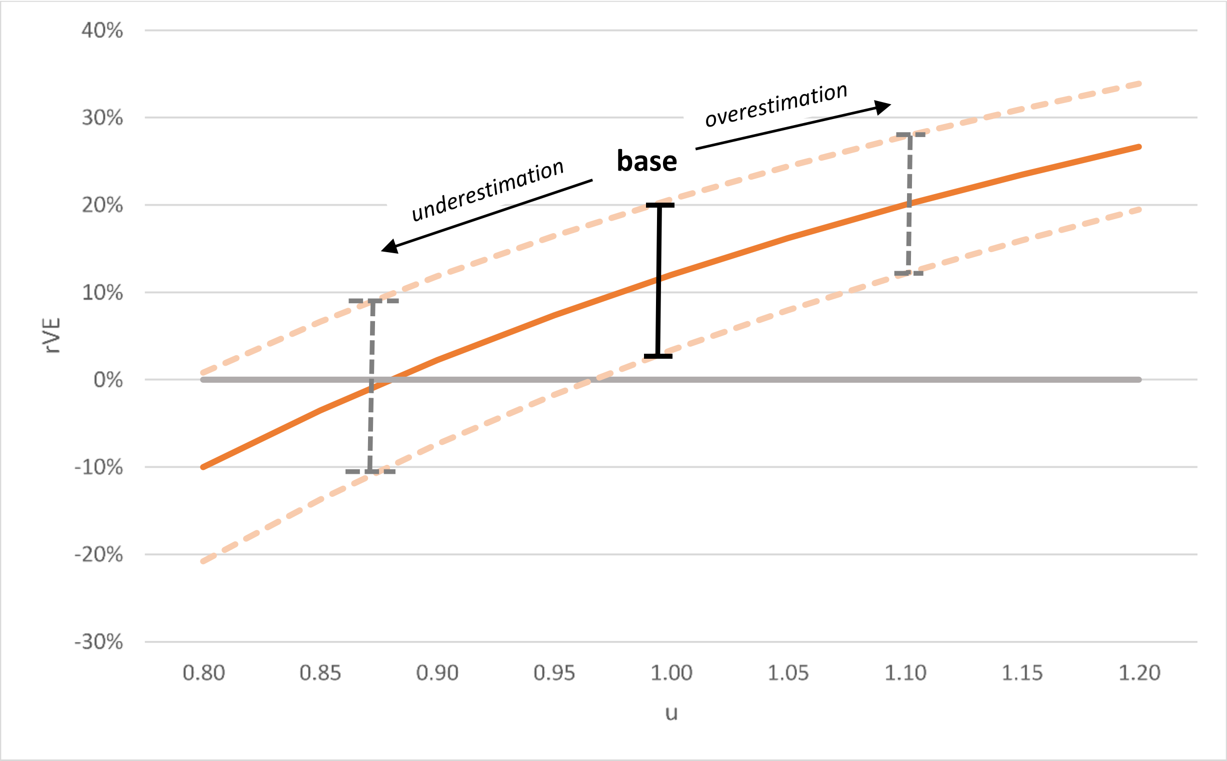

We were only able to match the cohort at the state level to address geographic heterogeneity in viral activity. Let us assume that within-state heterogeneity still confounded our results. If HD recipients more often lived in localities with severe viral activity than aIIV3 recipients (resulting in $u < 1$), PERR will underestimate the treatment effect. If the opposite is true ($u > 1$), PERR will overestimate the treatment effect.

References

1. van Aalst R, Gravenstein S, Mor V, Mahmud SM, Wilschut J, Postma M, Chit A. Comparative effectiveness of high dose versus adjuvanted influenza vaccine: A retrospective cohort study. *Vaccine*. 2020;38(2):372-9.
2. Lin NX, Henley WE. Prior event rate ratio adjustment for hidden confounding in observational studies of treatment effectiveness: a pairwise Cox likelihood approach. *Stat Med*. 2016;35(28):5149-69.
3. Tannen RL, Weiner MG, Xie D. Use of primary care electronic medical record database in drug efficacy research on cardiovascular outcomes: comparison of database and randomised controlled trial findings. *BMJ*. 2009;338:b81.
4. Yu M, Xie D, Wang X, Weiner MG, Tannen RL. Prior event rate ratio adjustment: numerical studies of a statistical method to address unrecognized confounding in observational studies. *Pharmacoepidemiol Drug Saf*. 2012;21 Suppl 2(S2):60-8.
5. Angrist JD, Pischke J-S. *Mostly harmless econometrics: An empiricist's companion*: Princeton university press; 2008.
6. Uddin MJ, Groenwold RH, van Staa TP, de Boer A, Belitser SV, Hoes AW, Roes KC, Klungel OH. Performance of prior event rate ratio adjustment method in pharmacoepidemiology: a simulation study. *Pharmacoepidemiol Drug Saf*. 2015;24(5):468-77.
7. Tannen RL, Weiner MG, Xie D. Replicated studies of two randomized trials of angiotensin-converting enzyme inhibitors: further empiric validation of the 'prior event rate ratio' to adjust for unmeasured confounding by indication. *Pharmacoepidemiol Drug Saf*. 2008;17(7):671-85.
8. Austin PC. Balance diagnostics for comparing the distribution of baseline covariates between treatment groups in propensity-score matched samples. *Statistics in medicine*. 2009;28(25):3083-107.
9. van Aalst R, Thommes E, Postma M, Chit A, Dahabreh IJ. On the causal interpretation of rate-change methods: the prior event rate ratio and rate difference. *American journal of epidemiology*. 2020.
